# Supplementary figures and images for: Characterizing patterns of diffusion tensor imaging variance in aging brains
Source: J Med Imaging (Bellingham). 2024 Aug 24;11(4):044007. doi: 10.1117/1.JMI.11.4.044007 (PMC11344569; doi:10.1117/1.JMI.11.4.044007)

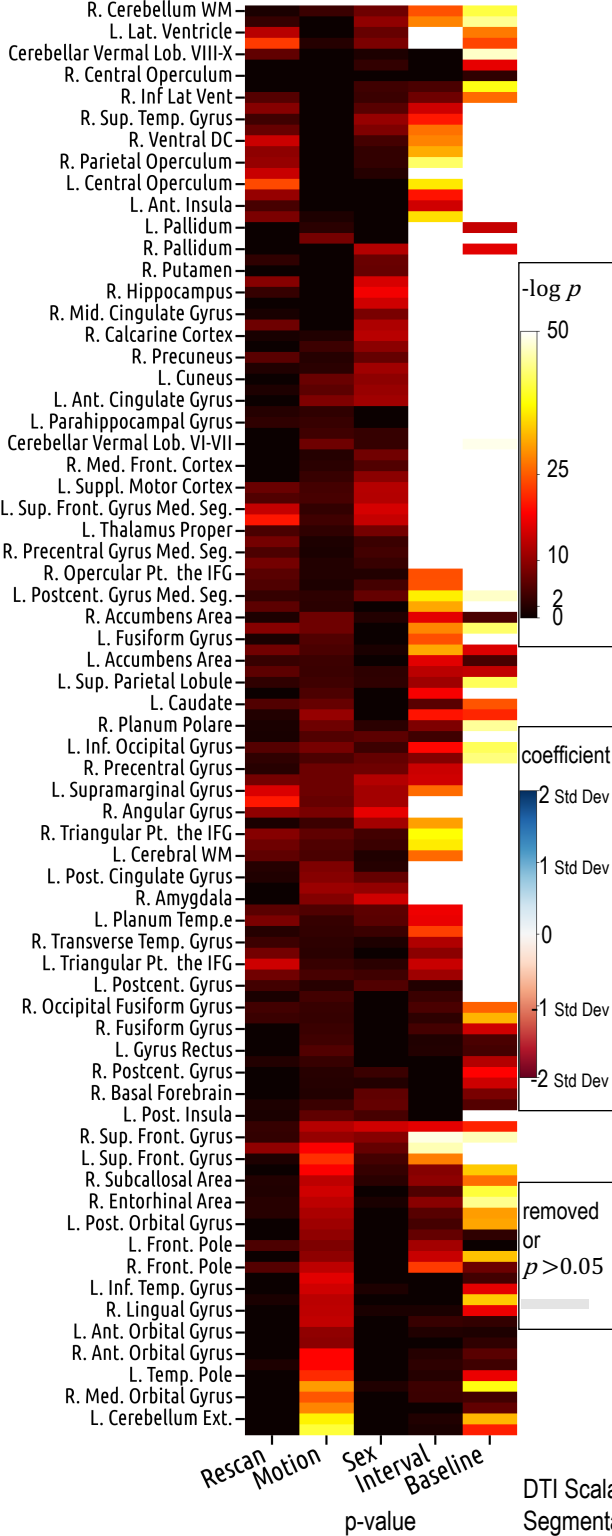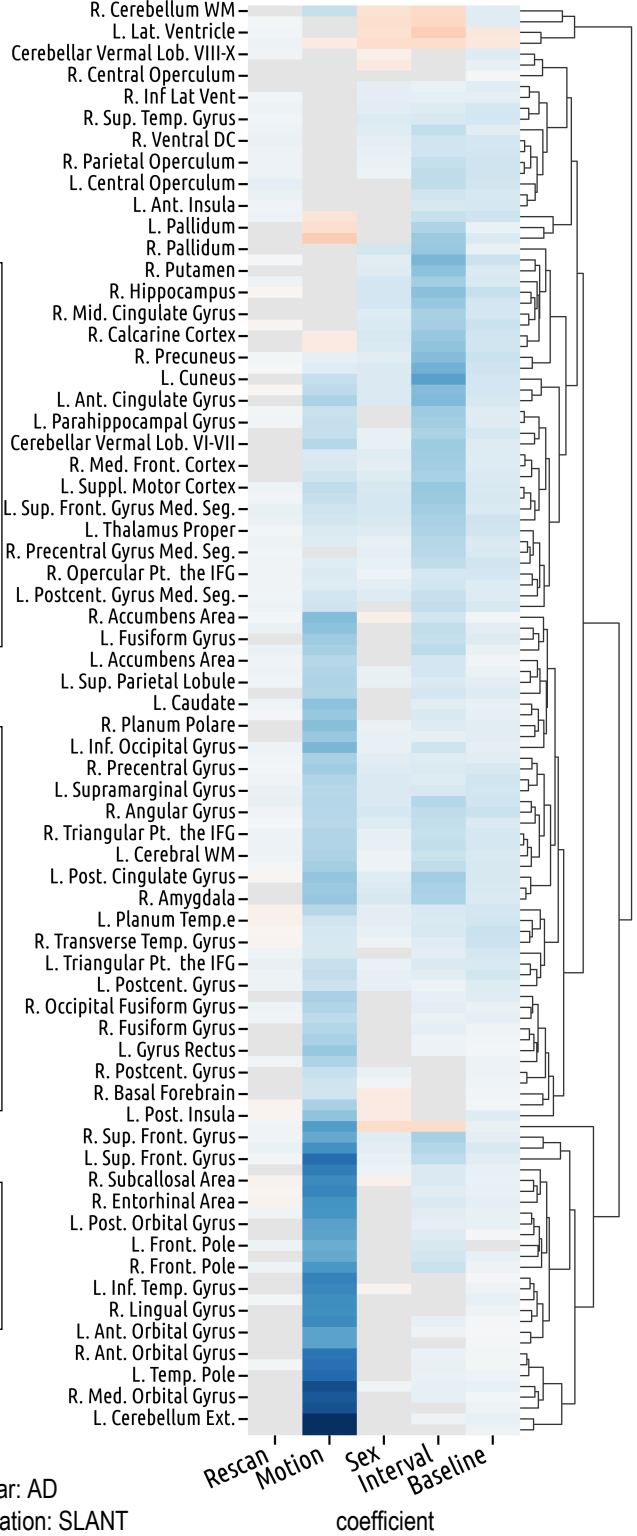

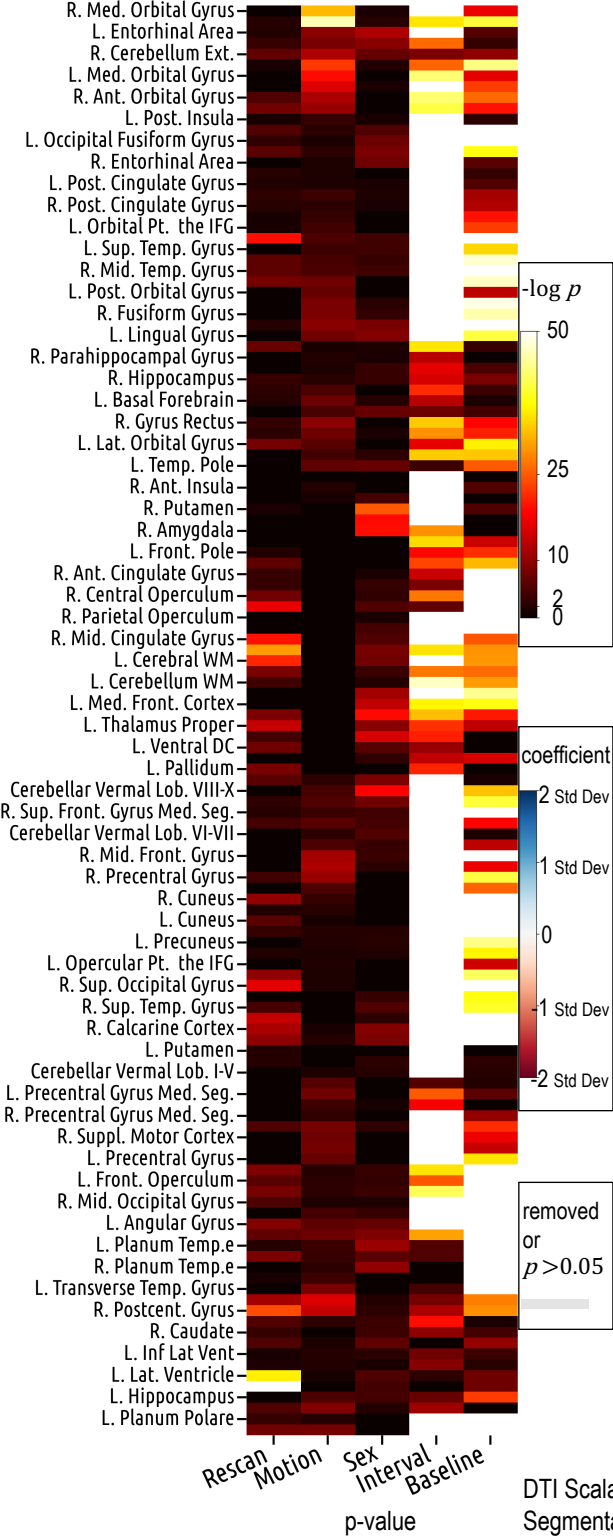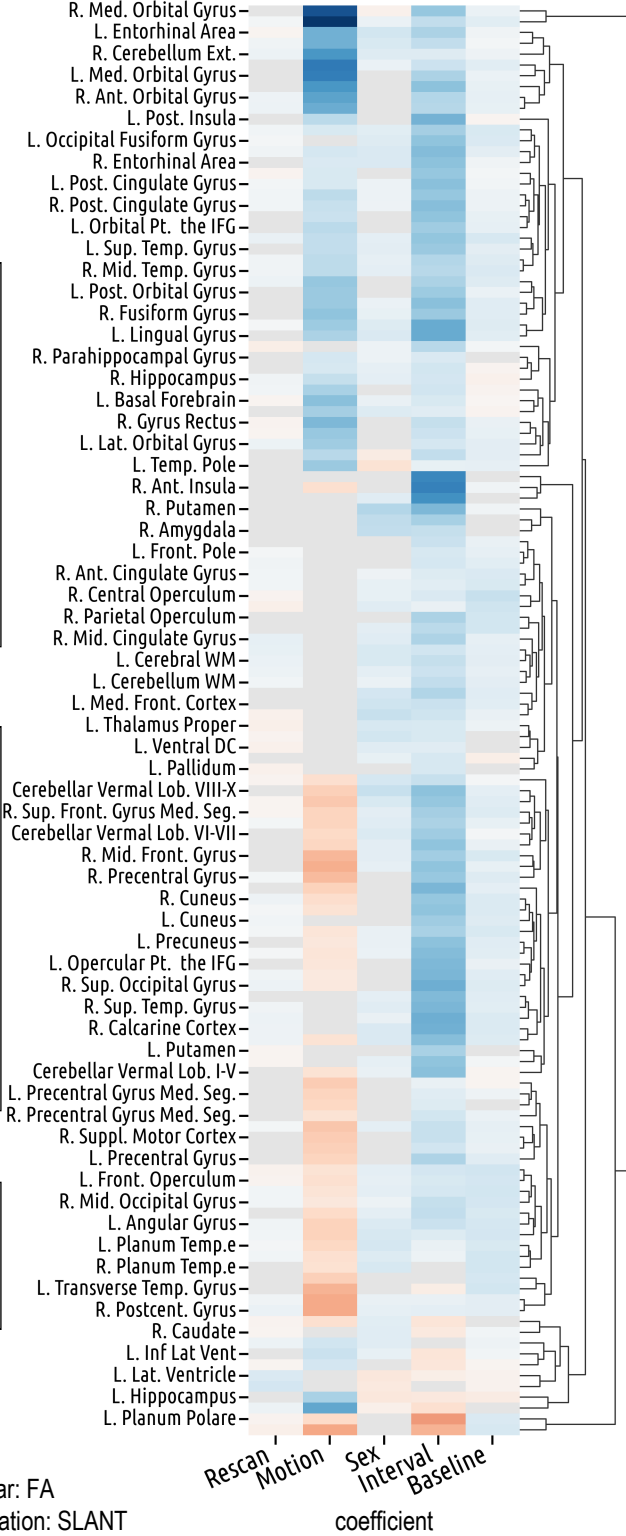

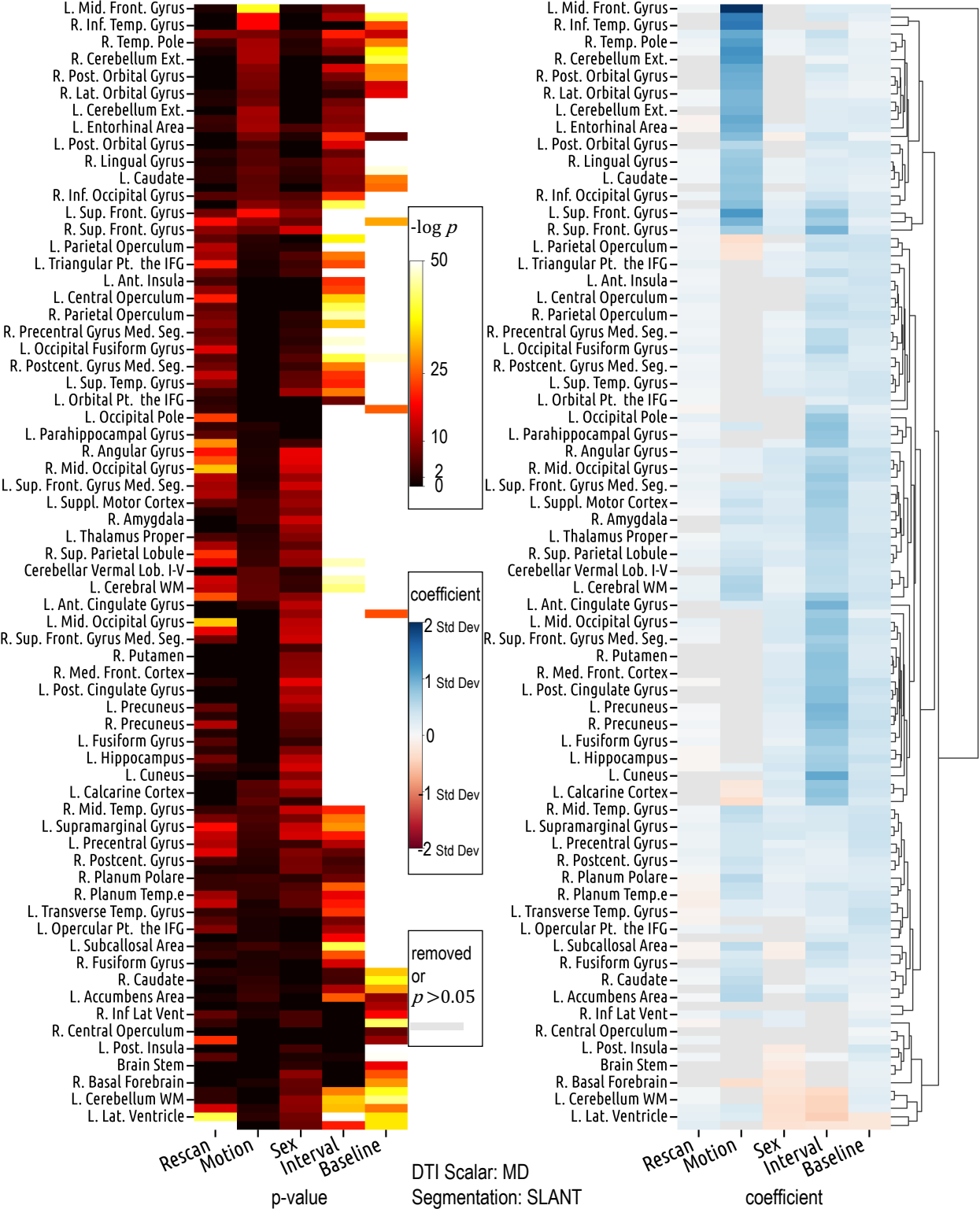

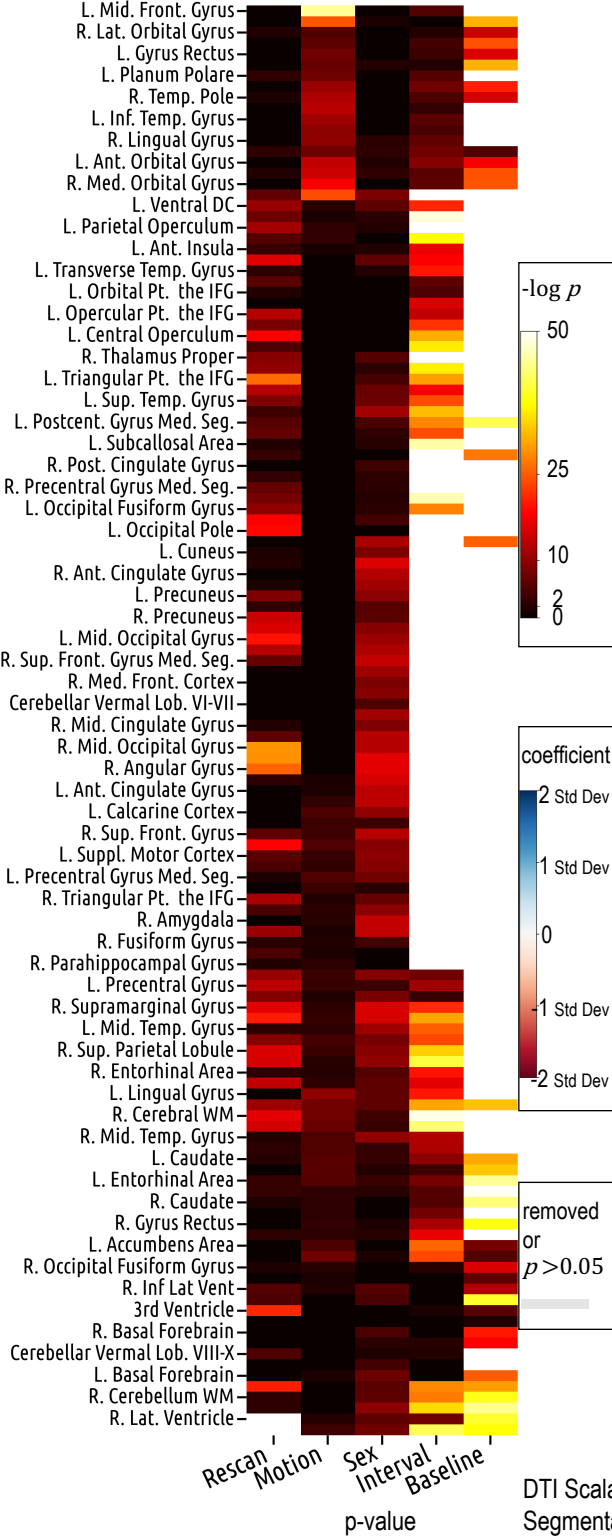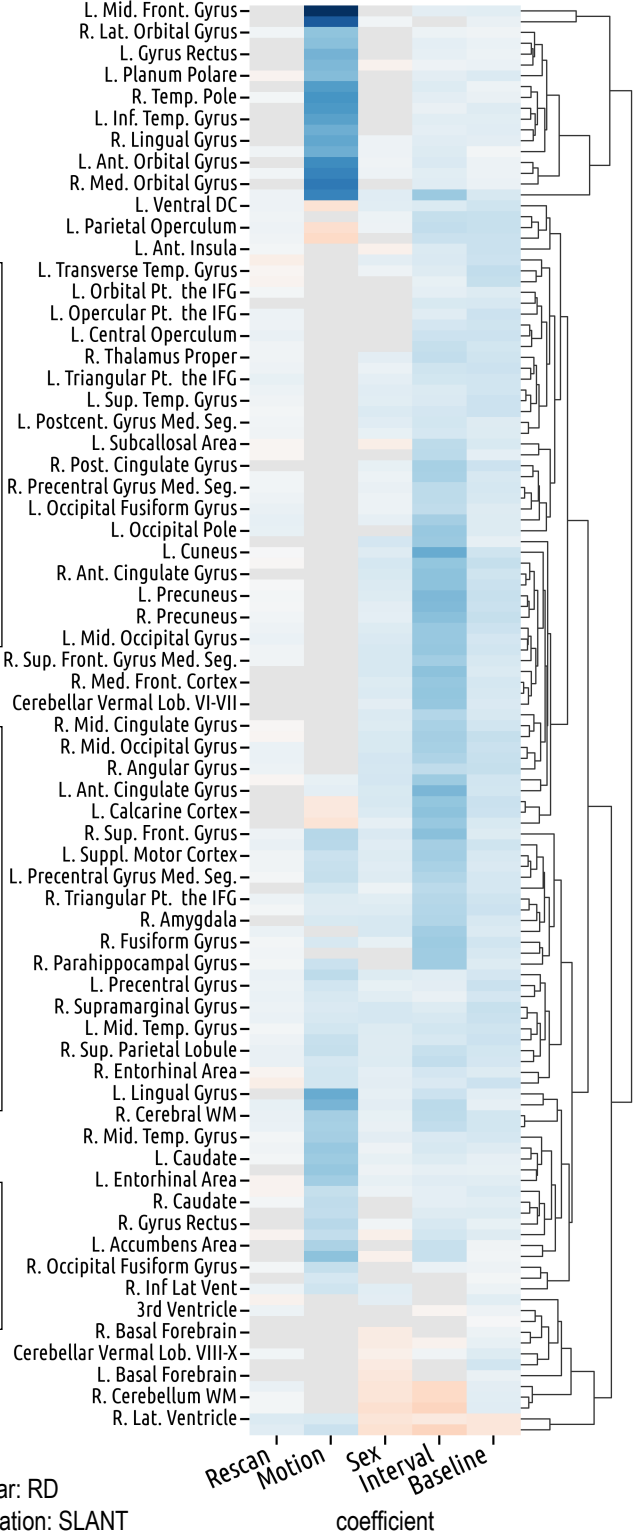

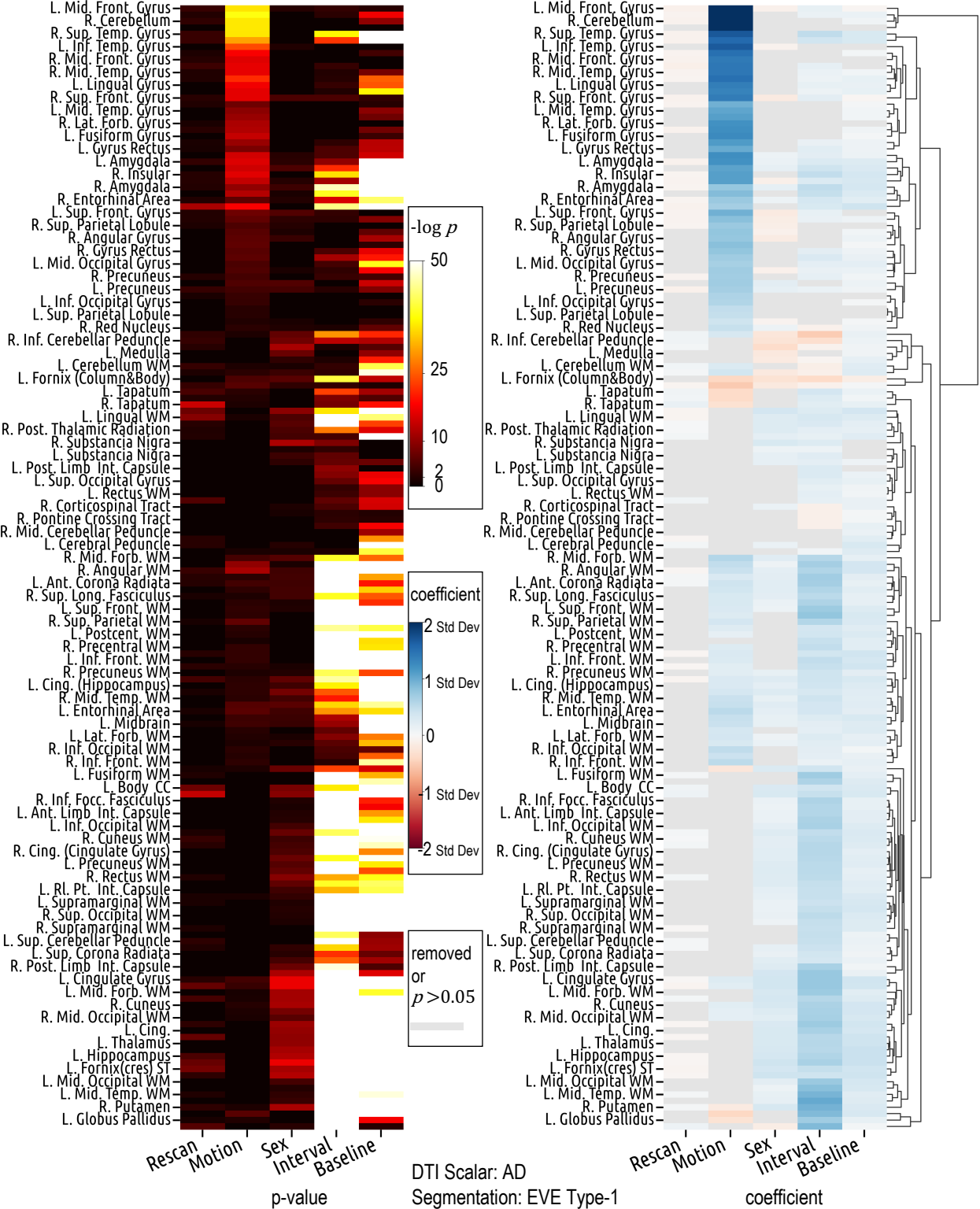

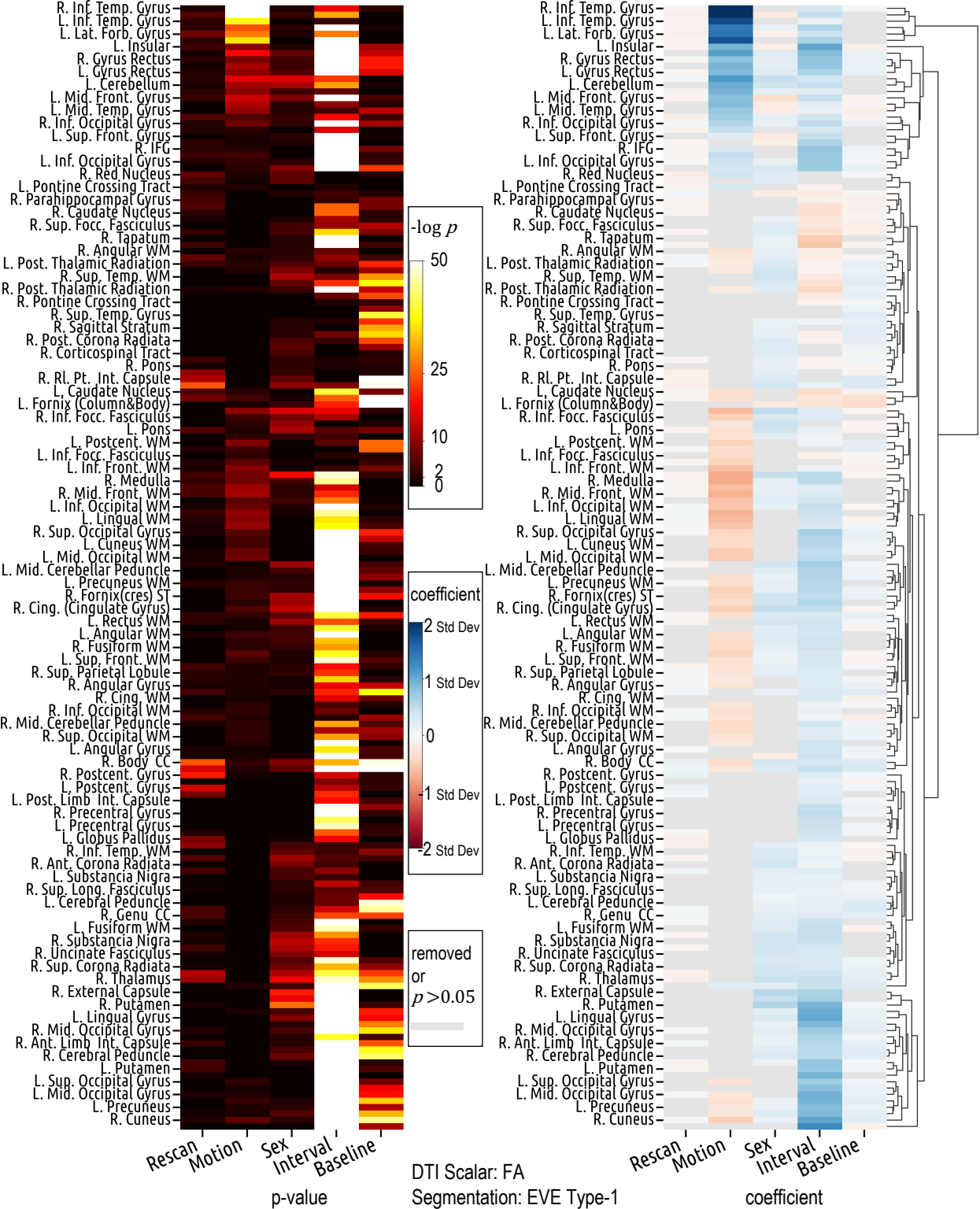

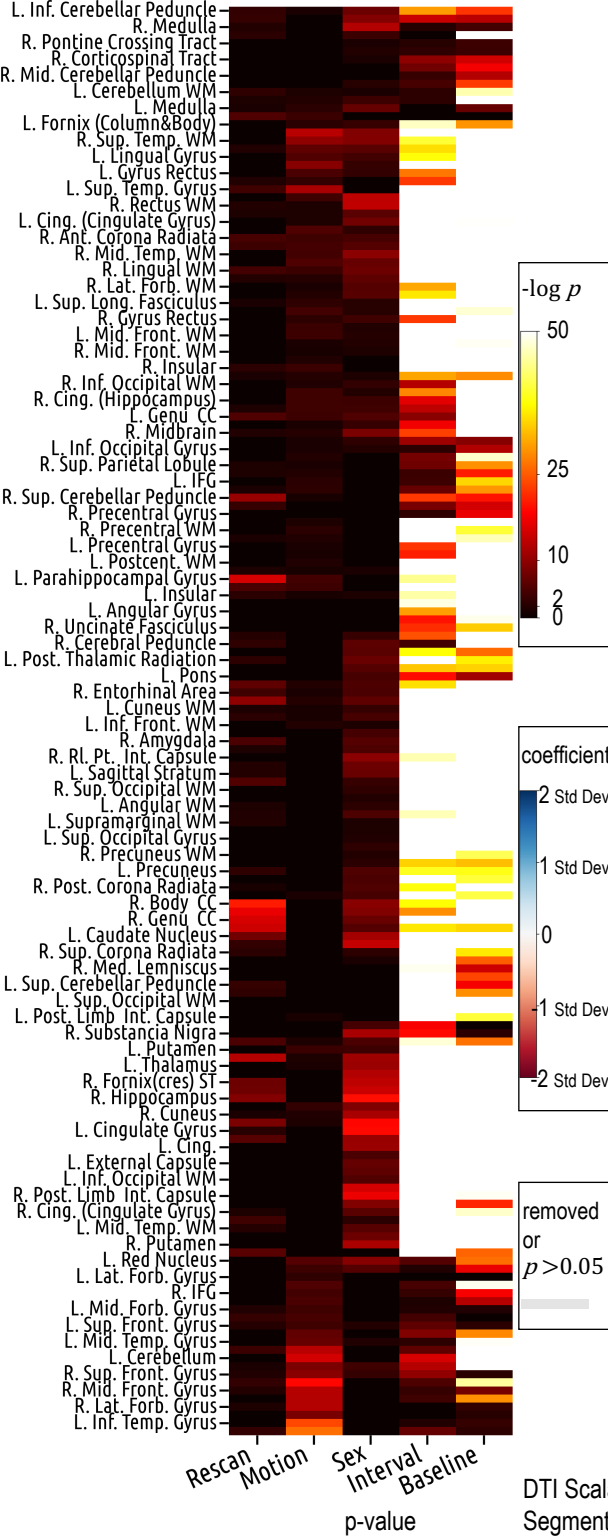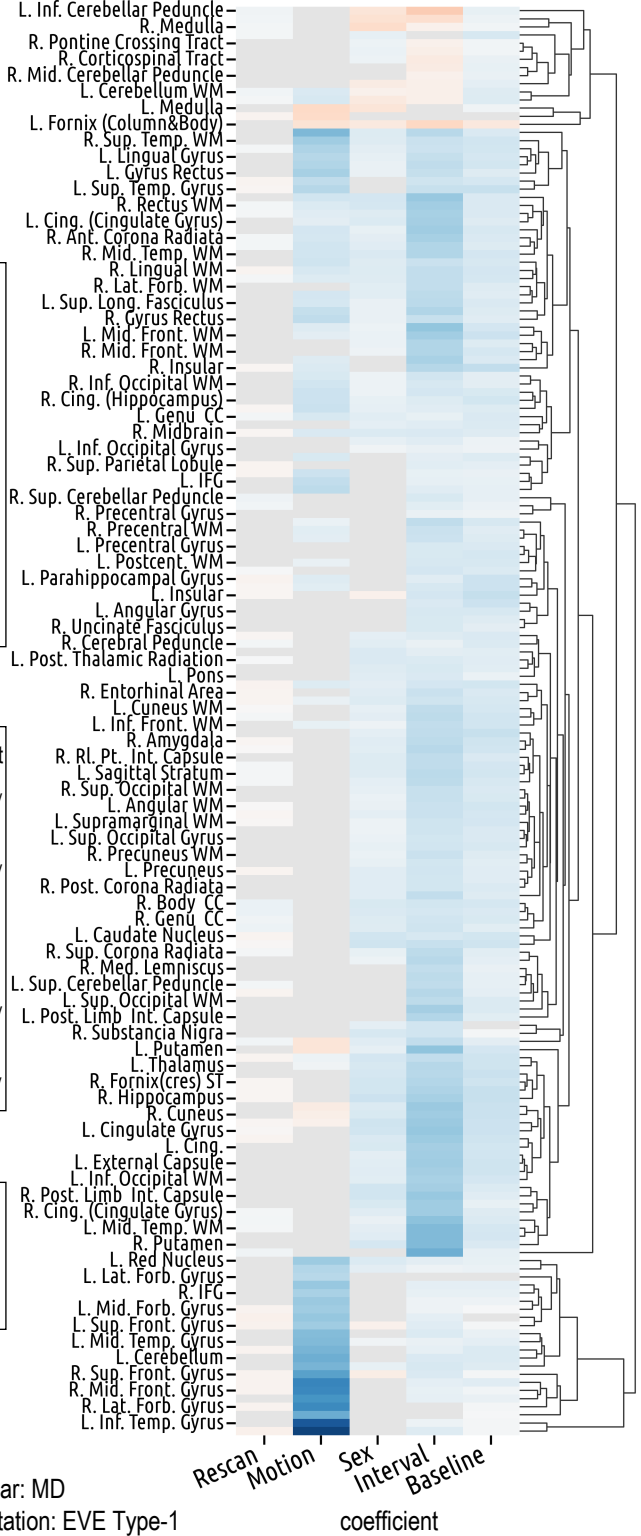

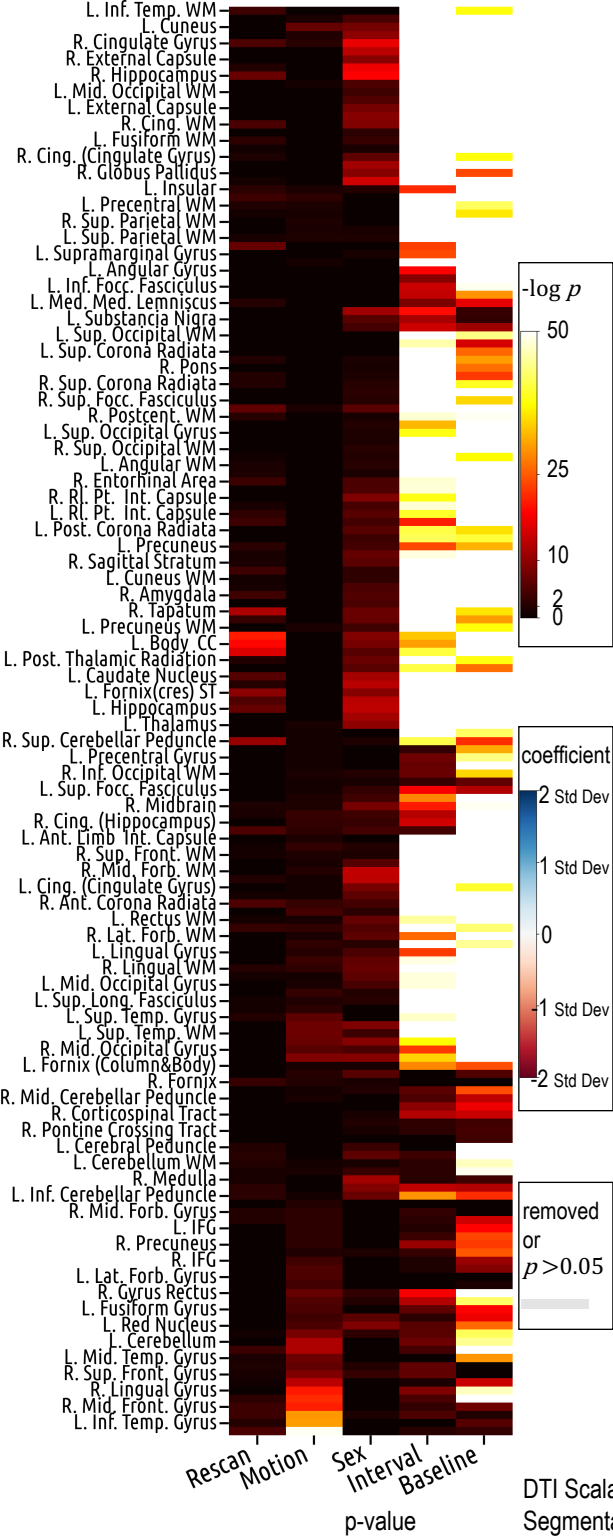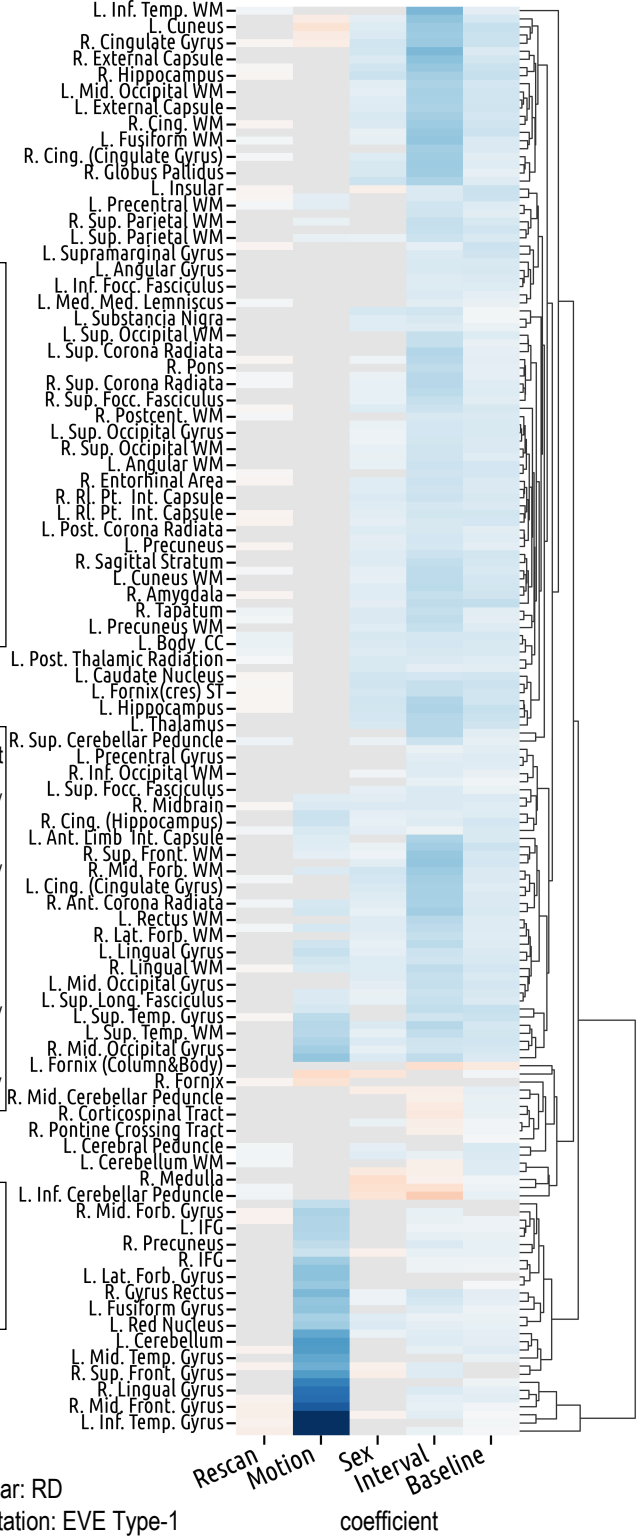

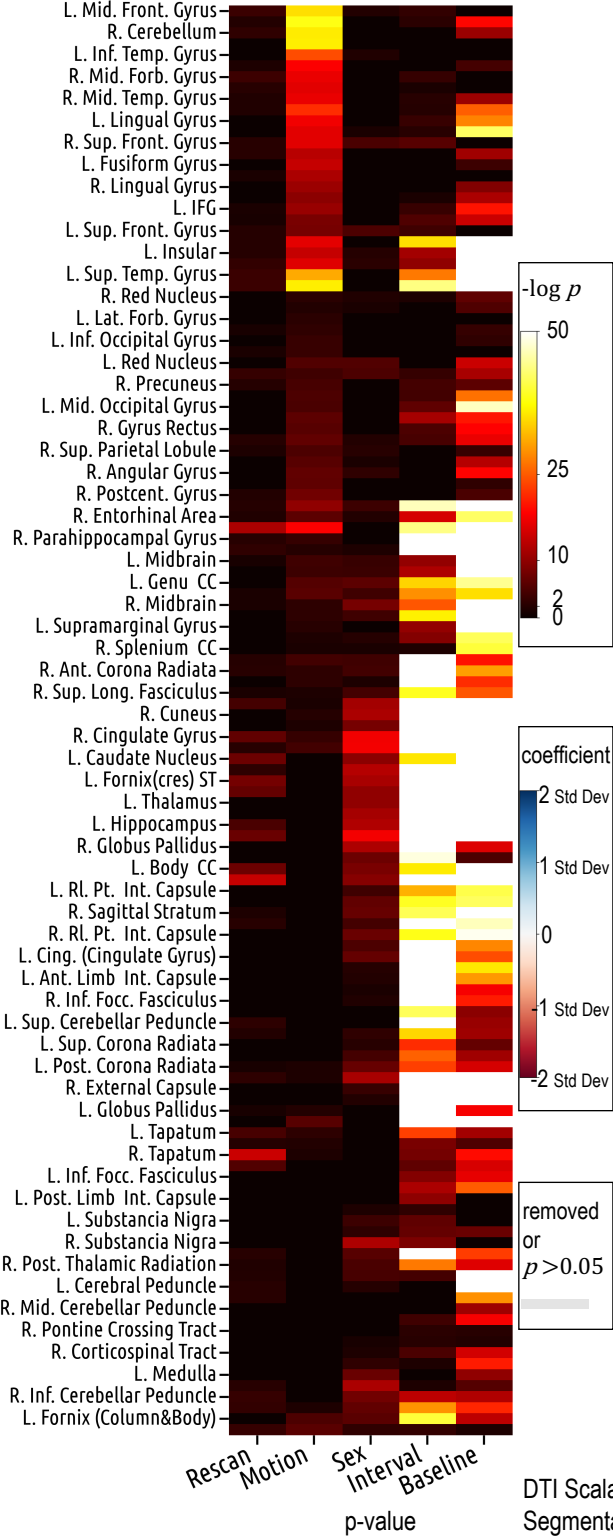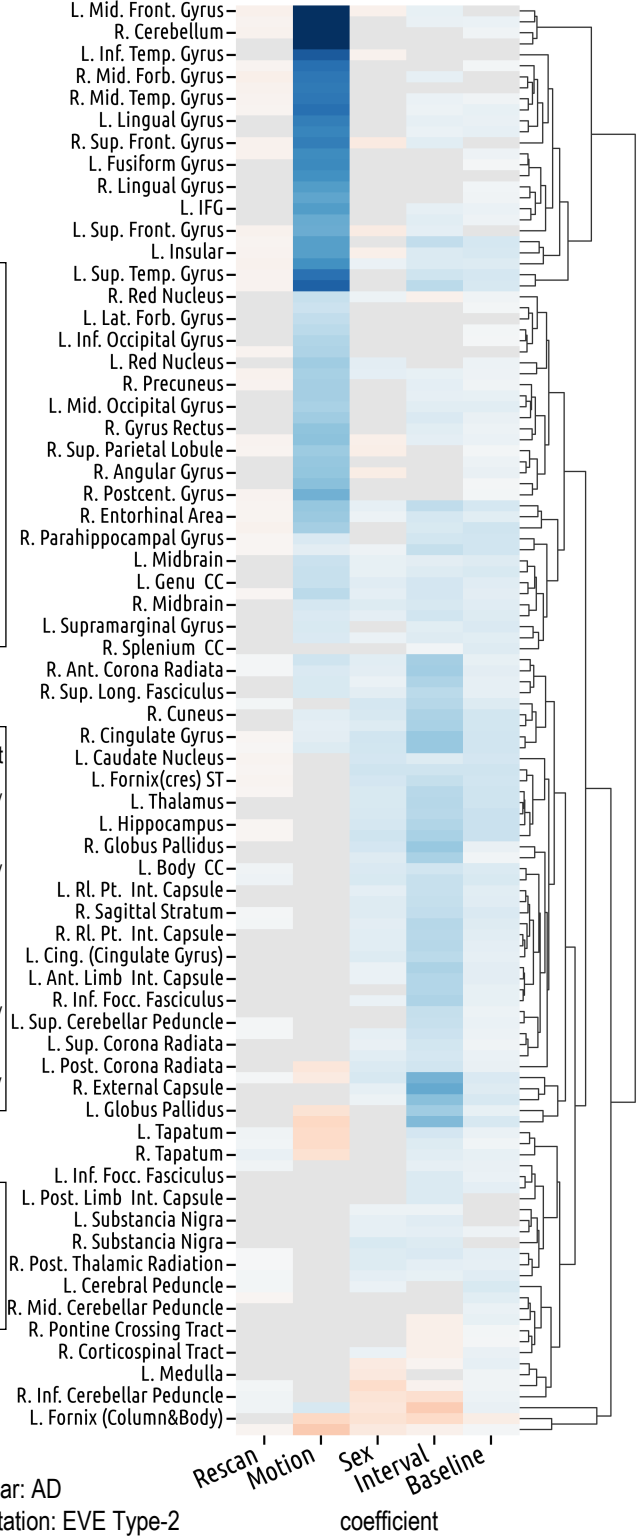

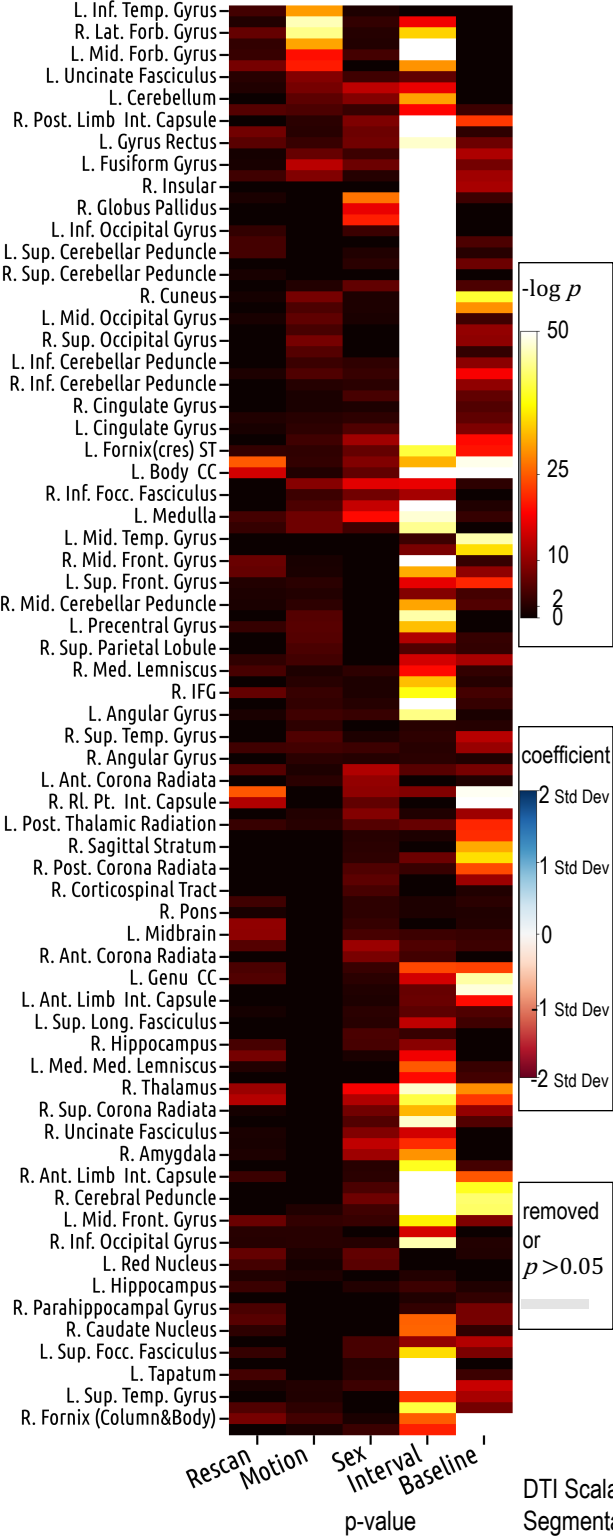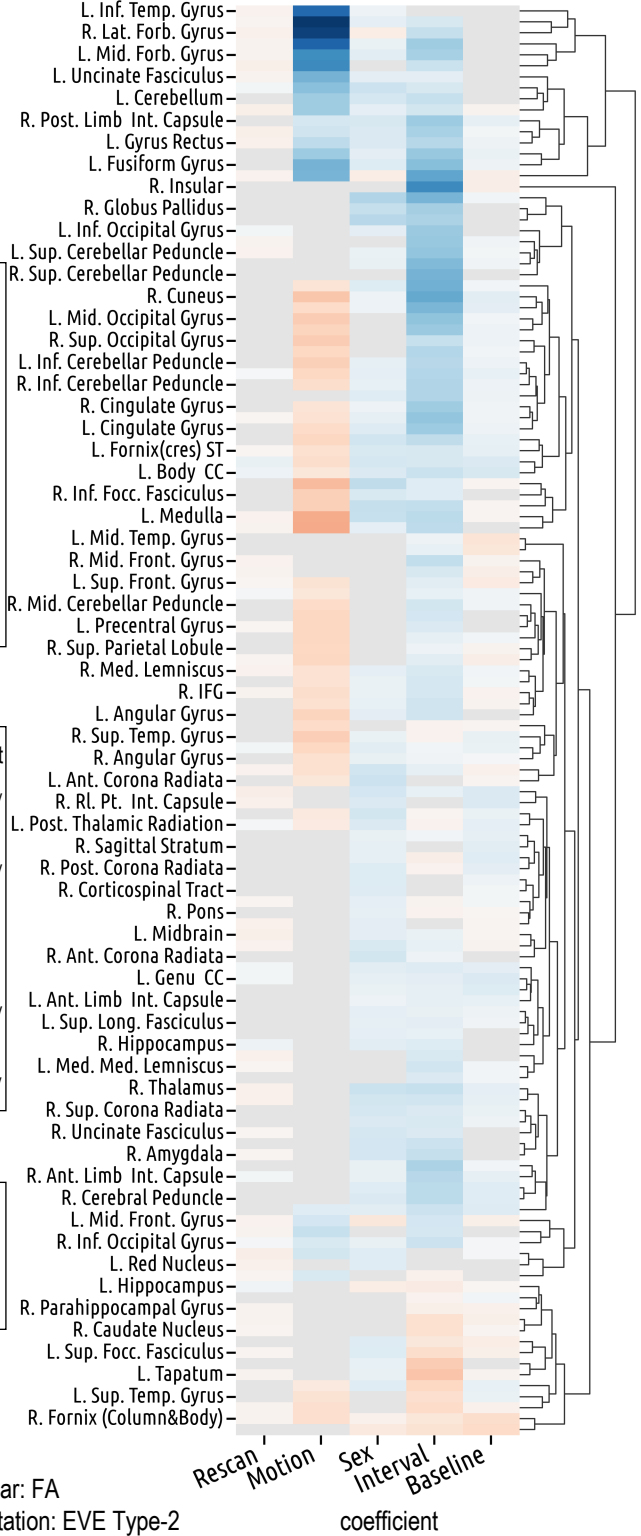

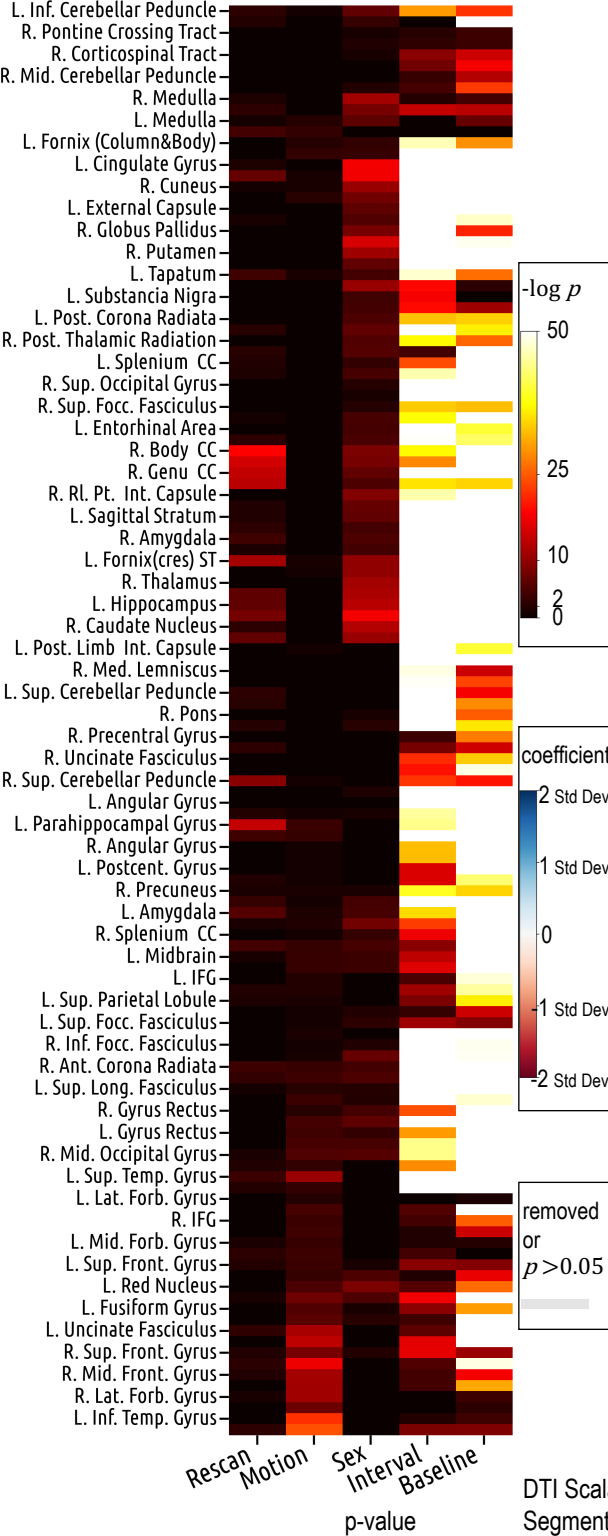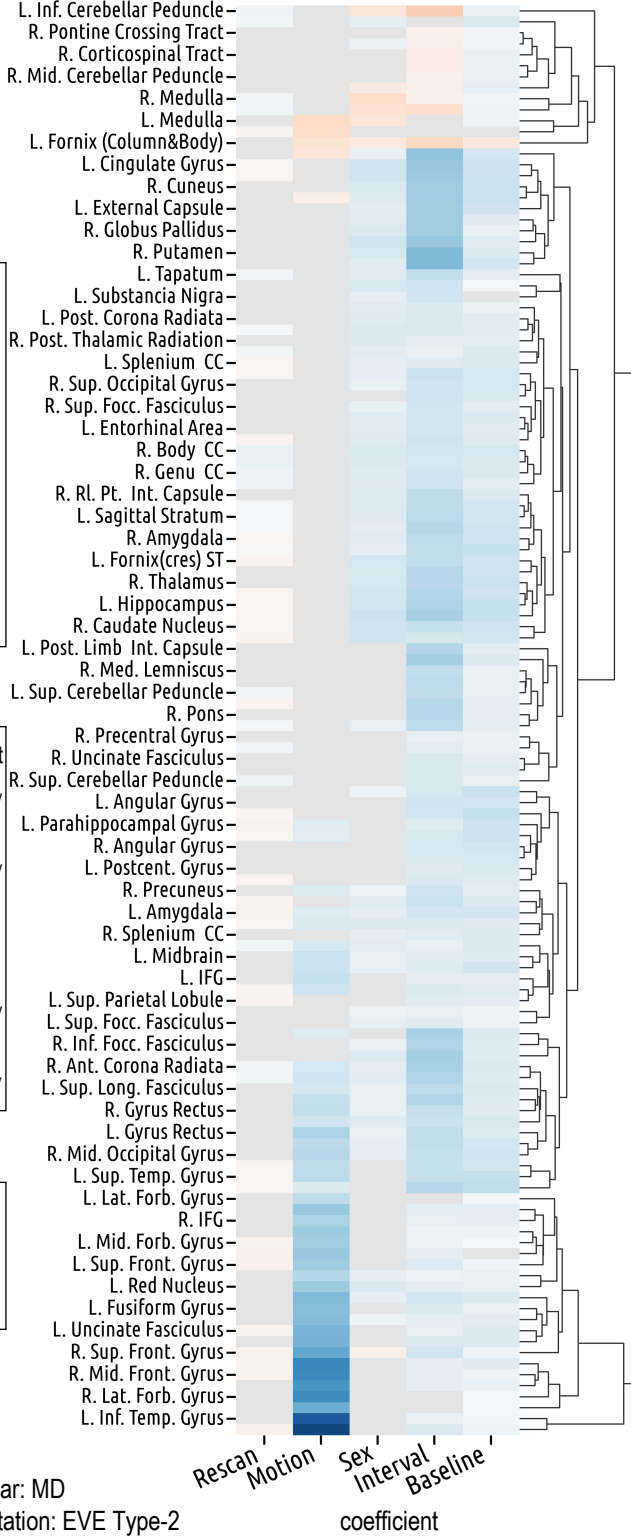

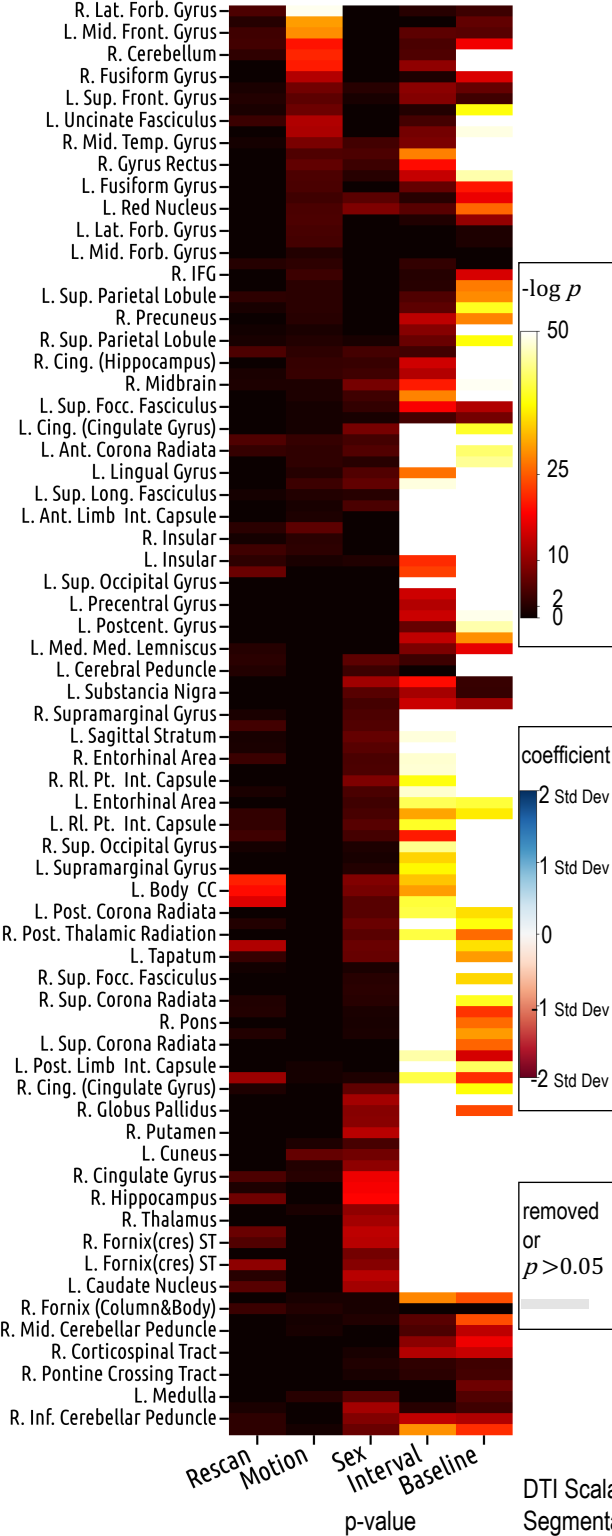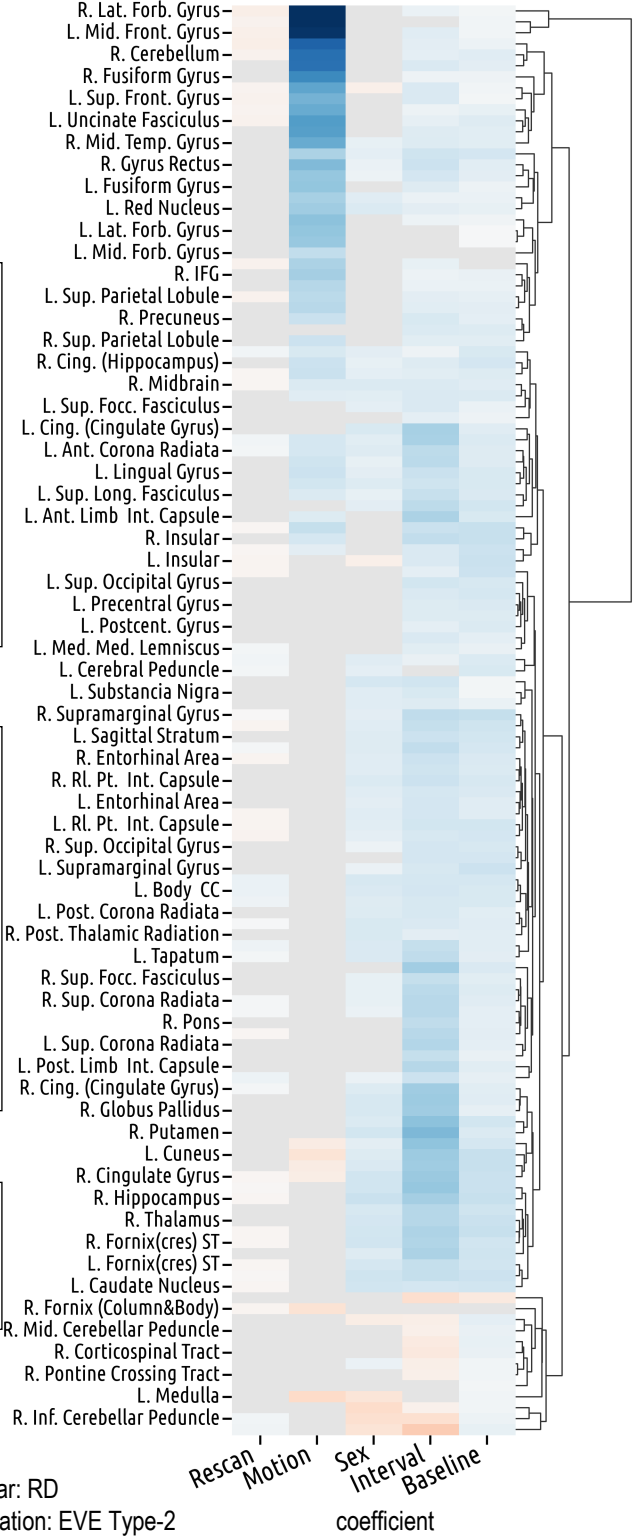

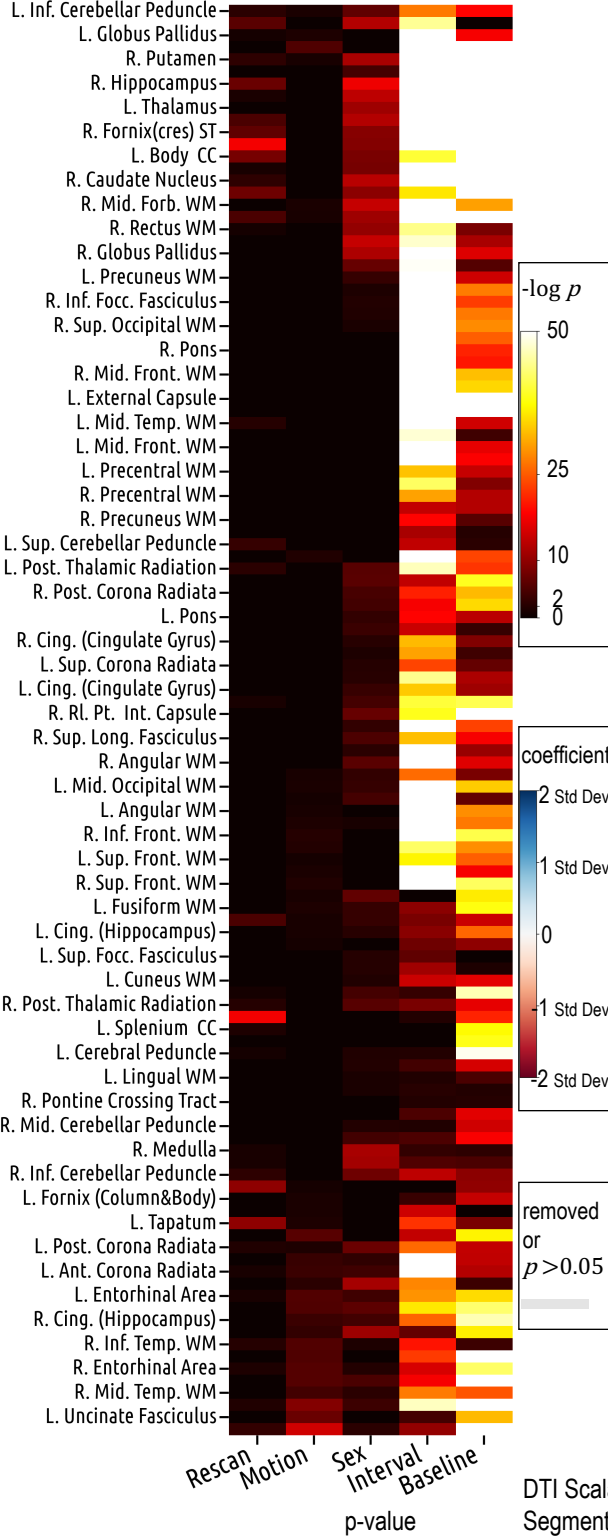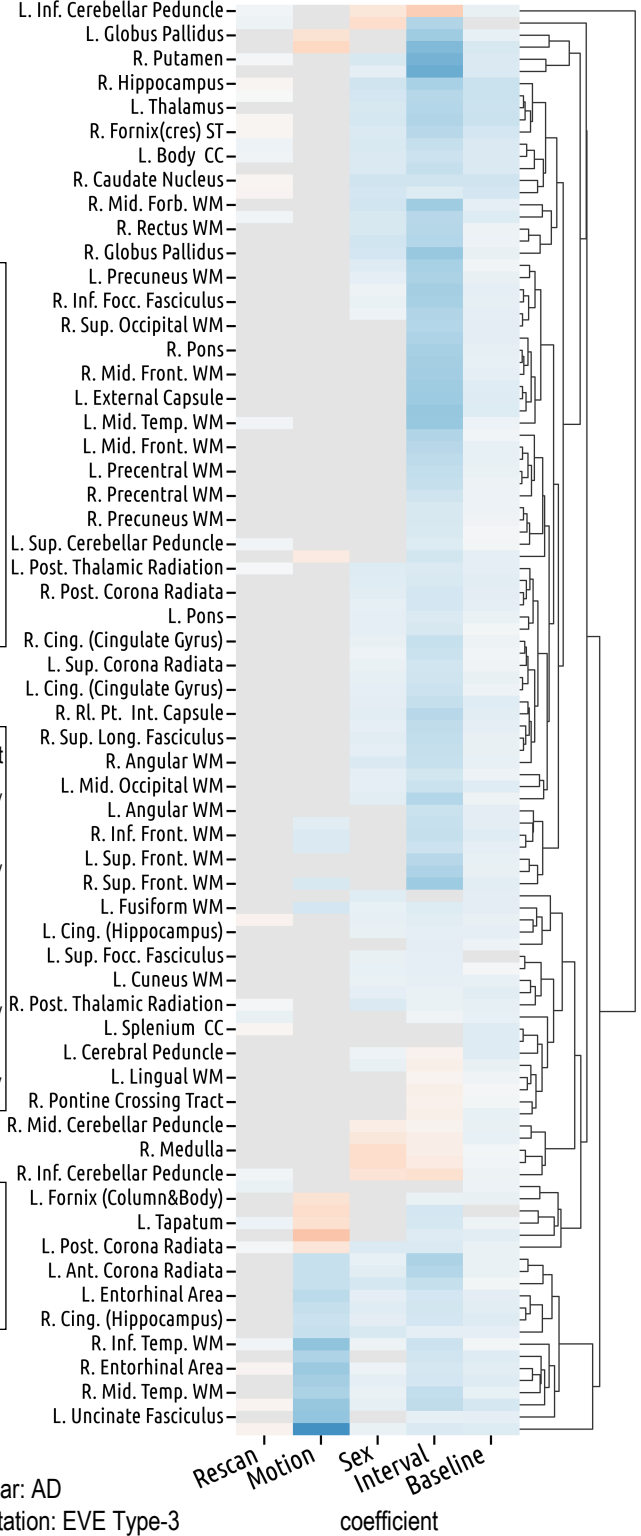

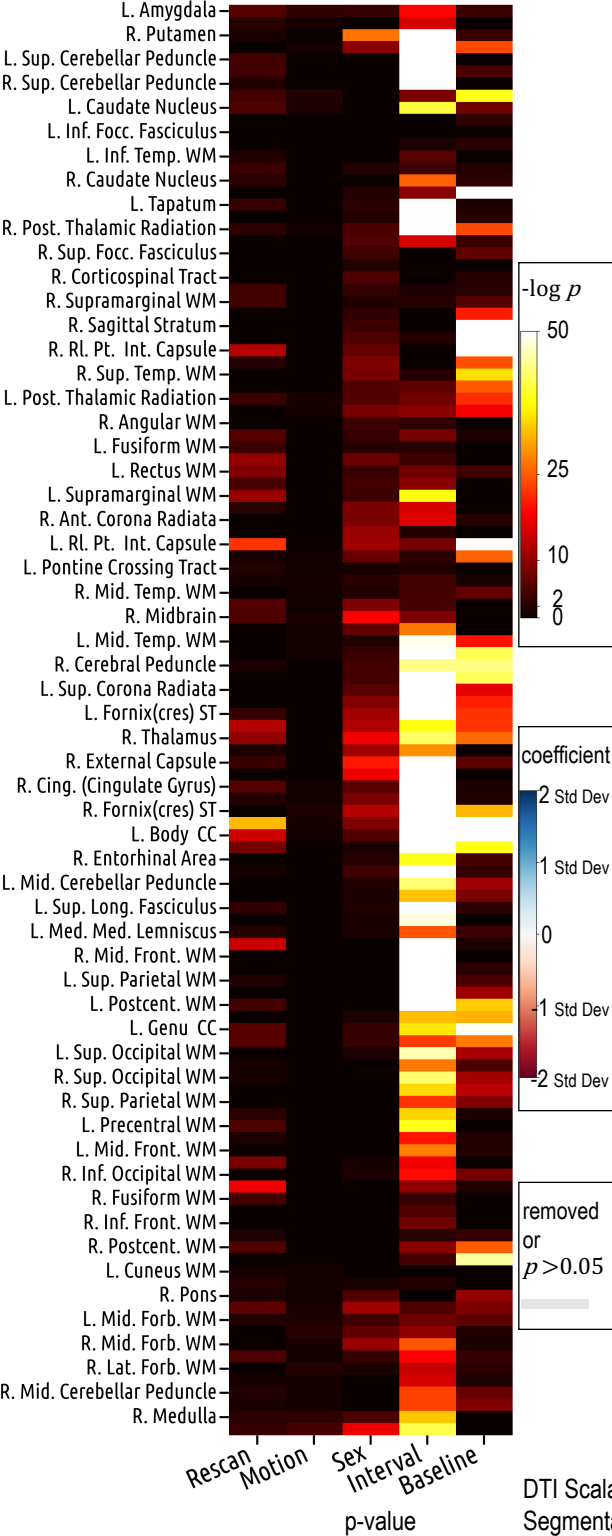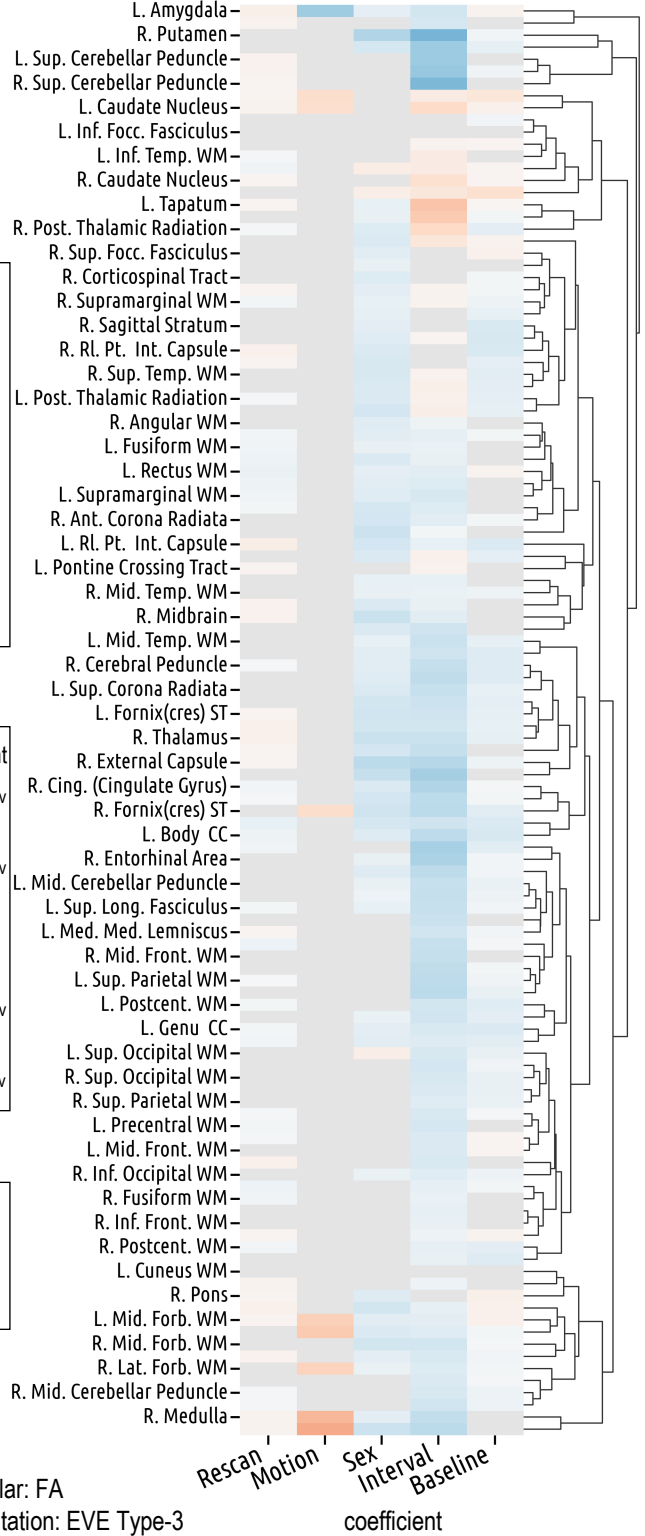

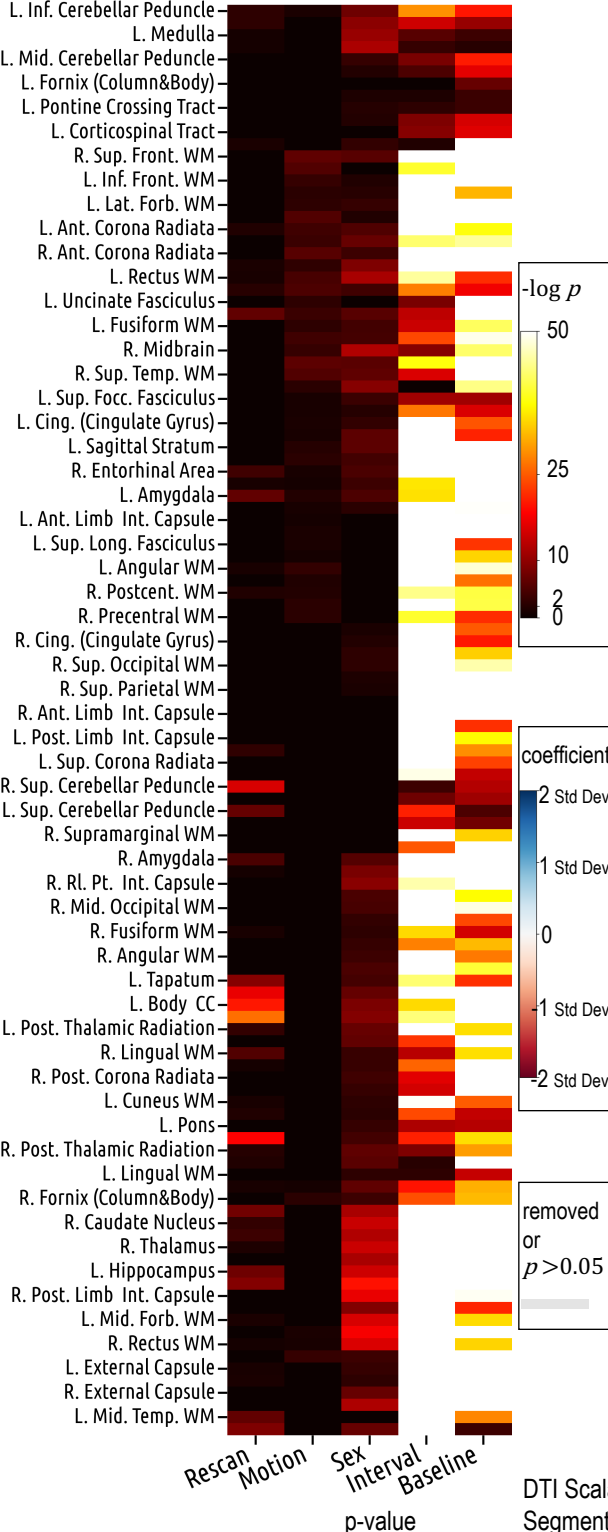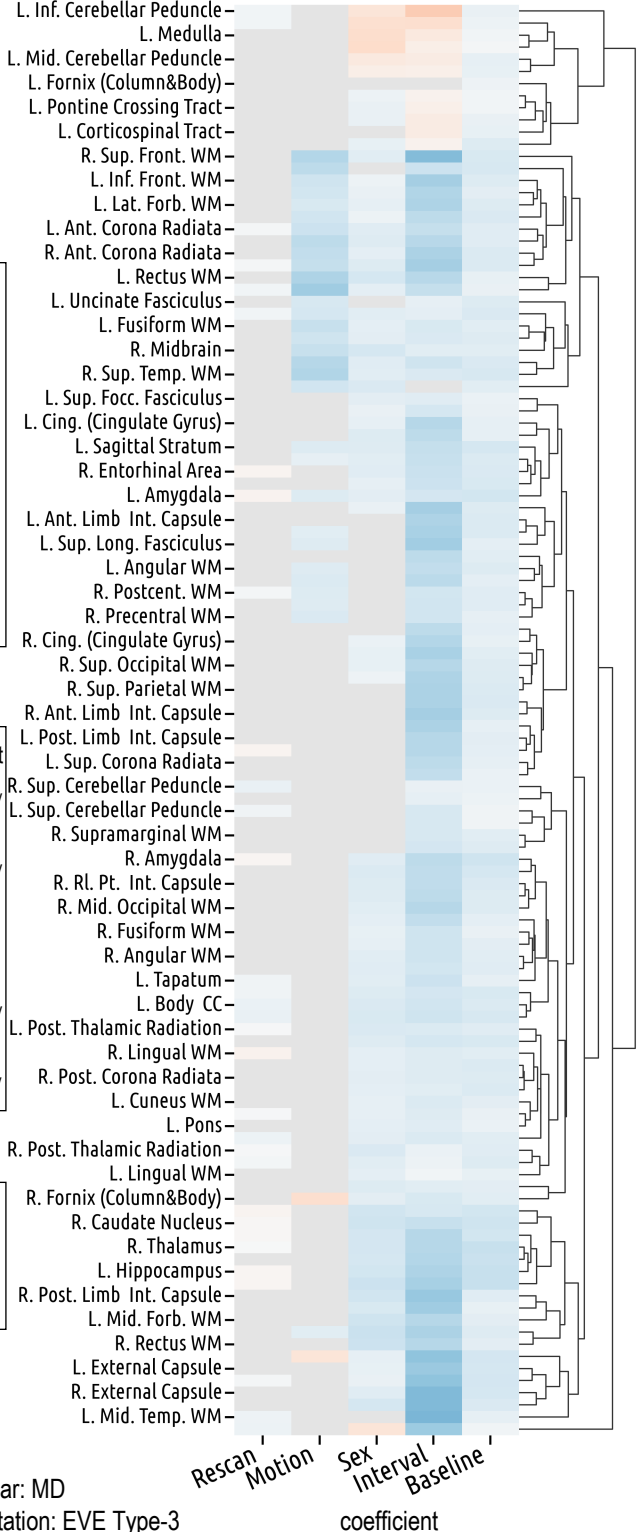

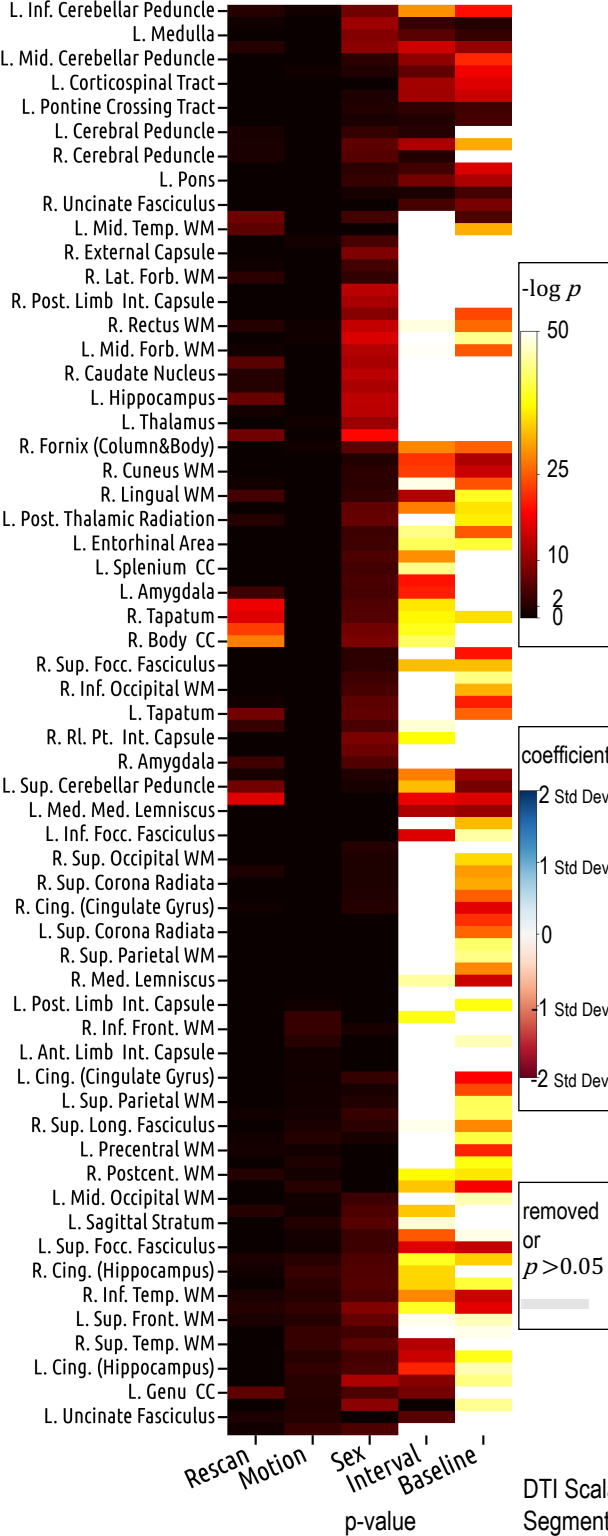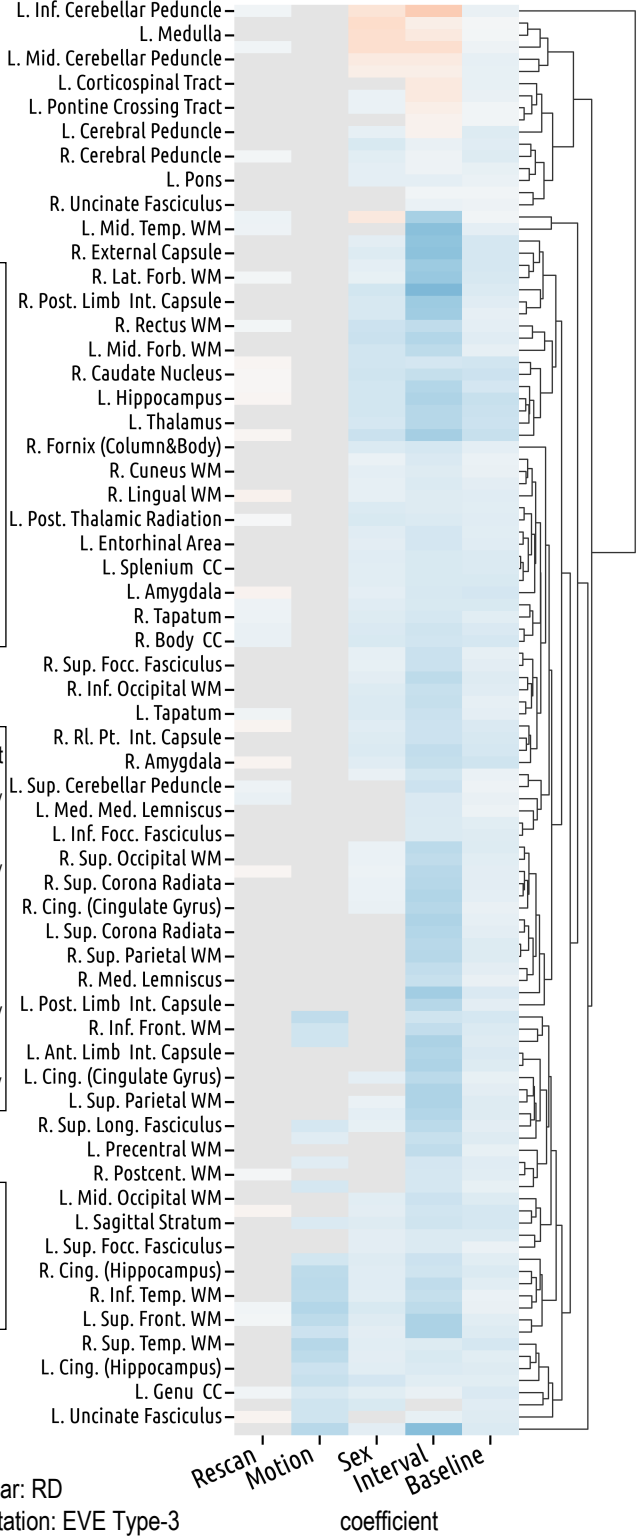

Supplement: Supplementary file 3 [file JMI_011_044007_SD003.pdf]
